# Authors' reply

**Published:** 2010

**Authors:** Vikas Khetan, Kshanada Gupta, Mohan E Ravindra, Gopal Lingam

**Affiliations:** Department of Vitreoretina and Ocular Oncology, Medical Research Foundation, Sankara Nethralaya, Chennai, India; 1 Department of Oculoplasty, Medical Research Foundation, Chennai, India

Dear Editor,

We are thankful to Chandravanshi *et al*.[1] for their keen interest and comments on our article, ‘Uveal melanoma presenting as staphyloma and cataract’.[2]

We agree that the histopathological diagnosis is confirmatory in the diagnosis of uveal melanoma and not magnetic resonance imaging (MRI). But the radiological features of uveal melanoma observed on MRI with T1 and T2 weighted images are very characteristic i.e. hyperintense on T1 and hypointense on T2. Further we believe that to label MRI as a confusing radiological tool in an aid to diagnose uveal melanoma needs a series of false positive reports and not a single case report study as cited by the author. The role of MRI as an aid to the diagnosis of uveal melanoma cannot be overemphasized.[3–6]

We also agree with the definition of staphyloma. But we would kindly like to bring to your notice that the photograph of the enucleated eyeball shows the near total loss of scleral fibers in the nasal quadrant while the superonasal region still shows few thinned, whitish scleral fibers. On slit-lamp examination it is the superonasal region (and not the complete nasal quadrant with near total loss of scleral fibers) which is accessible for examination. It clearly shows the bulging and thinning of sclera with uveal tissue seen through it. In case of an extrascleral extension, there is usually a breach in the sclera and the tumor grows outside the eye as well. In our case the entire tumor was still within the eye on gross examination. It was only histopathologically that an extrascleral extension could be found.

As far as we can gather from the re-read, we never implied origin of uveal melanoma in pre-existing blind eye. Our main intention was to stress the importance of radioimaging of blind eye as it may harbor uveal melanoma. We also agree with you that advanced uveal melanoma with retinal detachment and cataract are the likely causes of blindness in this case.

As mentioned in the article, the anterior segment examination of the other eye was within normal limits, including the intraocular pressure (one week later), gonioscopy and perimetry.

We appreciate your interest in the surgical technique of enucleation adopted in our case which we could not describe in detail as dictated by the word limit for the journal. Liberal lateral canthotomy, limited peritomy superonasally with gentle and meticulous dissection was carried out to prevent globe rupture. After removing the globe intact, the orbital tissue admixed with the orbital fat (lying in close proximity of the mass) was also excised.
